# Owning an Overweight or Underweight Body: Distinguishing the Physical, Experienced and Virtual Body

**DOI:** 10.1371/journal.pone.0103428

**Published:** 2014-08-01

**Authors:** Ivelina V. Piryankova, Hong Yu Wong, Sally A. Linkenauger, Catherine Stinson, Matthew R. Longo, Heinrich H. Bülthoff, Betty J. Mohler

**Affiliations:** 1 Department of Human Perception, Cognition and Action, Max Planck Institute for Biological Cybernetics, Tübingen, Germany; 2 Department of Philosophy of Neuroscience, Werner Reichardt Centre for Integrative Neuroscience, Tübingen, Germany; 3 Department of Psychology, Lancaster University, Lancaster, England; 4 Department of Psychological Sciences, Birkbeck, University of London, London, England; 5 Department of Brain and Cognitive Engineering, Korea University, Seoul, Korea; Royal Holloway, University of London, United States of America

## Abstract

Our bodies are the most intimately familiar objects we encounter in our perceptual environment. Virtual reality provides a unique method to allow us to experience having a very different body from our own, thereby providing a valuable method to explore the plasticity of body representation. In this paper, we show that women can experience ownership over a whole virtual body that is considerably smaller or larger than their physical body. In order to gain a better understanding of the mechanisms underlying body ownership, we use an embodiment questionnaire, and introduce two new behavioral response measures: an affordance estimation task (indirect measure of body size) and a body size estimation task (direct measure of body size). Interestingly, after viewing the virtual body from first person perspective, both the affordance and the body size estimation tasks indicate a change in the perception of the size of the participant's experienced body. The change is biased by the size of the virtual body (overweight or underweight). Another novel aspect of our study is that we distinguish between the physical, experienced and virtual bodies, by asking participants to provide affordance and body size estimations for each of the three bodies separately. This methodological point is important for virtual reality experiments investigating body ownership of a virtual body, because it offers a better understanding of which cues (e.g. visual, proprioceptive, memory, or a combination thereof) influence body perception, and whether the impact of these cues can vary between different setups.

## Introduction

Immersive virtual environments (VEs) have great potential as interactive mediums for performing rehabilitation treatments and therapies in a controlled manner [1]–[3]. The users of such applications are often represented by avatars in virtual reality (VR). The avatars are usually human-like stylized characters, which may be presented to the user in 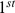 person perspective (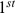 PP) or 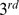 person perspective (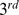 PP). This does not necessarily mean that the person represented by the avatar in VR immediately embodies the avatar. However, for many VR applications to be effective, people need to identify themselves with their self-representing avatar and feel ownership over its virtual body. In this study, we investigate the conditions required for embodying a virtual avatar. We also introduce novel measures to assess embodiment of a virtual avatar.

Although people normally only feel a sense of ownership over their own body and its parts, illusions of body ownership can make people perceive non-corporal objects such as artificial limbs as parts of their own body [4]–[8]. Research suggests that this embodiment of artificial limbs can be divided into several subcomponents, including senses of ownership, agency and location [7]–[9]. Three main types of response measure are used to quantify the effect of body ownership: 1) subjective measures, including self-report questionnaires [8], [10]–[13], 2) physiological measures, including heart-rate [10] or skin conductance [12], [14] and 3) behavioral measures, including matching tasks [14], proprioceptive drift [15], or size drift in perceived size of body parts [11], [16].

Most investigations of body ownership employ the rubber hand illusion (RHI) paradigm [5], [6] (see [7] for an overview). Generally in RHI experiments, the participant sits with their hands resting on a table in front of them. One of the participant's hands is blocked from view, and a rubber hand is positioned on the table between the unseen hand and the midpoint between the participant's hands. A sense of ownership over the rubber hand can be induced by applying synchronous visual-tactile stimulation by simultaneously touching or stroking the seen rubber hand and the unseen real hand [6], [7]. Synchronous multisensory stimulation over one body part can even extend to ownership over the entire body [17]. Also, it has been shown that multisensory illusions can be induced with synchronous visual-motor stimulation without the need for passive touch [4], [18], [19]. However, if the visual-tactile stimulation is asynchronous (the visual stimulation is not synchronous with the touch or the stroking), a sense of ownership is typically not induced [7]. In addition, the RHI only occurs if the posture of the rubber hand and the participant's own hand match and they are congruently positioned [6], [20].

Recently, due to the flexibility that VEs provide for modifying the appearance and the shape of the body, many researchers have begun to investigate the sense of body ownership in immersive VEs [10], [11], [16], [21], [22]. Some scientists suggest that in a VE, it is not only visually and spatially synchronous touch that can induce a body ownership illusion, but also synchronous sensory-motor stimulation [11], [16], head tracking [10], or seeing the body from 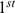 PP [10], [23].

### Background: measuring embodiment and body perception in VR

VEs have been used to induce a sense of body ownership in several different experiments. Lenggenhager et al. [15] used the RHI paradigm in a VR experiment to induce an illusion of body ownership that involves the whole body. Petkova and Ehrsson [12] showed that their participants experienced a body swap with the experimenter after seeing themselves shake hands with the experimenter from the experimenter's perspective. Slater et al. [10] demonstrated that male participants can feel ownership over a virtual female avatar. Their findings suggest that 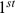 PP and synchronous touch are important factors for inducing the illusion of body ownership in VEs [10]. Other researchers have found that body ownership, combined with the sensory information that the participant perceives in the VE, has an impact on the felt size of certain body parts, body shape, and body symmetry [11], [14], [16]. Normand et al. [11] showed that participants perceive their belly to be bigger after synchronous visual-tactile stimulation. Van der Hoort et al. [14] used legs which were significantly different in size from the legs of their participants and found that after synchronous visual-tactile stimulation, participants felt ownership over the legs. The sense of ownership over the different sized legs also had an impact on the perceived size and distance of objects in the environment [14]. Kilteni et al. [16] provided synchronous sensory-motor stimulation of a virtual hand which was a of a considerably different length than participants' real hands, and found that participants experienced ownership over the virtual hand. This previous research provides evidence of the usefulness of VR in exploring the plasticity of body representation.

### Background: using affordances to measure body size perception

People can adapt very quickly to changes in their body's dimensions, even when their bodies are artificially modified [24]. Several researchers suggest that changes in the width and the size of the body can influence decisions and estimations about affordances [24], [25]. Gibson defined affordances as the relationship between an organism's action capabilities and the environment [26]. Thus the concept of affordances is used as a connection between the person's perceived body (in terms of its action capabilities) and environmental constraints. For instance, when someone intends to perform an action, such as passing through an aperture like a doorway, the way the action is performed is influenced by the person's perceptions of their affordances [27]. Affordance perception is typically measured by having individuals estimate their ability to perform in a specific environmental setting. For example, perceptions of affordances for aperture pass-ability are assessed by having individuals estimate whether they can pass through apertures of various widths. A wealth of research has shown that humans are extremely accurate in their perceptions of affordances, even when the action capabilities of their bodies change [24], [25], [28]. For example, if one's body width increases, individuals are capable of quickly adjusting their judgments of whether they can pass through an aperture accordingly. In order to be able to pass through an aperture, people need a space at least as wide as their body [25]. Thus, the width of the aperture depends on the width of the body. For this reason, affordances judgments can be used as an implicit measure to indirectly access information about the person's perceived body size.

### Background: traditional methods of measuring body size perception

Perceived body size is an aspect of body image [29], [30]. Body image is the mental, multi-modal perceptual, conceptual, or emotional representation of one's own body, which involves sensory inputs perceived through 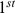 person experience and through the experience of the body as an object in the physical world [29]–[32]. Body size, as an assessment of body image has already been investigated in various ways (see [33] for an overview of the literature). There are a variety of response measures, such as figure rating scales [33]–[35], drawing of own body [36], optical distortion techniques [37], [38], behavior matching, and affordance measures [39] (see [40] for an overview) used for body size estimations.

### Aim of our research

**Figure 1:**
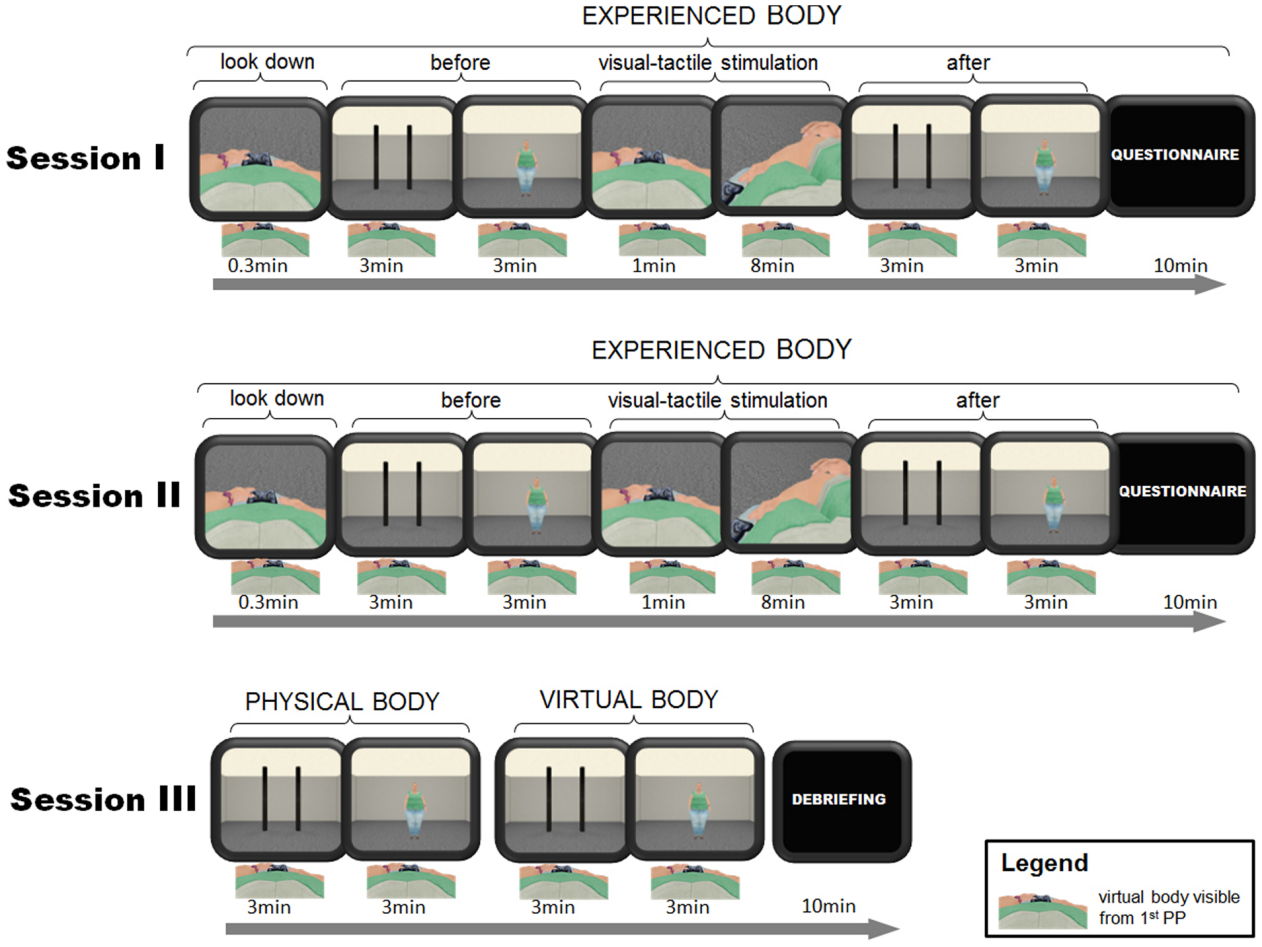
Schema illustrating the experiment.

Considering previous research, we investigated body ownership using an immersive VE. Specifically, we wanted to assess whether women could experience ownership over a virtual body that is considerably smaller or larger than their physical body that was viewed from 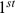 PP (see Figure 1). To gain a better understanding of the mechanisms underlying body ownership, we used an embodiment questionnaire. We also introduced two new behavioral response measures for estimating body ownership: an affordance estimation task (indirect measure of body size) and a body size estimation task (direct measure of body size). Our aim was to find out whether these two measures can be used as reliable measures for body ownership.

Another novel aspect of our research, not found in the existing literature, is that we distinguished between the 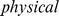, the 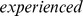 (note, that 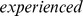 body is a term which we use only in the paper for marking a distinction; the term was not used in any communication with our participants) and the 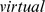 body:

• the 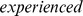 body - the body that the participant feels she has at that moment
• the 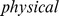 body - the participant's own body
• the 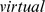 body - the body that the participant sees when she looks down in the VE at the place where she expects her physical body to be

The aim of this threefold distinction is to offer a better understanding of which cues (e.g. visual, proprioceptive, memory, or a combination thereof) influence the participants' body perception. Additionally, the impact of these cues may vary between different setups, which is important for VR experiments investigating body ownership of a virtual body. For instance, in the VE presented on a head-mounted display (HMD) the participants no longer have visual information about their own physical body. Instead, they receive visual information about a virtual body. However, they still receive somatosensory, proprioceptive and memory cues from their physical body. Thus, it is important to know how the cues that influence body perception interact and to what extent each of these cues affects the participants' body perception. The three bodies that we consider in our study tap into different cues that may influence body perception in VR:

• the 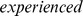 body - a combination of visual, proprioceptive and memory cues
• the 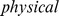 body - proprioceptive and memory cues
• the 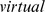 body - visual cues

**Figure 2:**
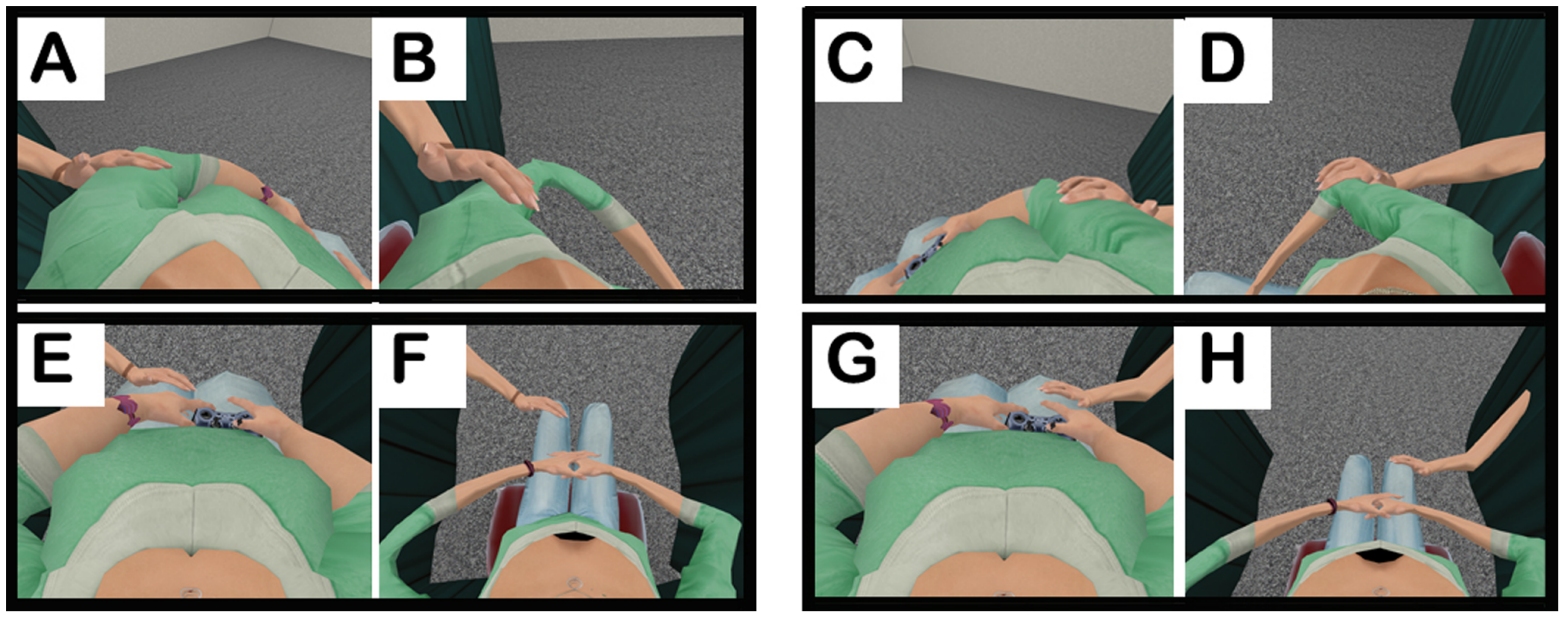
Perspective of the participant during visual-tactile stimulation. The animated virtual hand reaches through the black curtain to stroke the left arm (A - overweight, B - underweight), the right arm (C - overweight, D - underweight), the left leg (E - overweight, F - underweight) and the right leg (G - overweight, H - underweight) of the 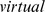 body.

In our experiment each participant was first asked to provide affordance and body size estimations (always in this order) for the body that the participant feels she has at that moment (the 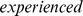 body) before and after the visual-tactile stimulation (synchronous or asynchronous - in a counter balanced order) (e.g Session I and Session II - the only difference between Session I and Session II was the type of visual-tactile stimulation) (see Figure 1 and Figure 2). Then at the end of the experiment in Session III, each participant performed the affordance task followed by the body size estimation task (no visual-tactile stimulation was involved) first for the 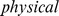 body and then for the 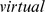 body. For these tasks, participants were specifically instructed.

We predicted that after synchronous visual-tactile stimulation the participants would experience increased ownership over the virtual body. Thus, their reports about the 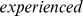 body would be biased by the size of the virtual body and they should experience a corresponding change in the perceived dimensions of their body. Additionally, we expected that, overall, body size estimations would be underestimated compared to the affordance estimations, because Warren and Whang, 1987, showed that in order for the gap to afford passing, the size of the aperture should be 1.3 times the size of their widest body part [25]. Therefore affordance estimations provide indirect measure of body size, in particular body width.

## Materials and Methods

### Ethics

The experiment was approved by the ethical committee of the Eberhard Karls University, Tübingen and written informed consent was obtained from all participants. Anonymized data collected for this experiment will be made available upon request.

### Technical Setup

The participants' head motions were tracked using an optical motion tracking system (16 Vicon MX13 cameras and Vicon Tracker 1.2 software). Thus, the participants received visual stimuli from a camera perspective that was updated to their head position and orientation (synchronous head motion), during the entire experiment including both the synchronous and the asynchronous stimulation. This was to prevent the participants from having motion-sickness due to asynchronous visual feedback [41].

The virtual scene was projected through a stereoscopic wide field of view (FOV) HMD - nVisor SX111 with 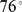 horizontal and 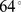 vertical FOV per eye. Since it has been shown that measuring and adjusting the IPD for each participant does not significantly improve participant's perception in HMDs up to 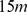 [42], we used the average IPD (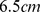) for all participants. The weight of the HMD was approximately 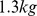. (See [43] for more information about the effects related to the weight of the HMD.) All participants were wearing the same HMD during the experiment, therefore any effect of the weight of the HMD on the participants' estimations should be the same for all participants in both the underweight and the overweight condition.

The average end-to-end latency of the described network (i.e., motion capture system, processing the captured data, and streaming the processed data to update the scene projected in the HMD accordingly) was approximately 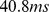 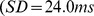). The end-to-end latency was measured using photodiodes as proposed by Di Luca [44]. The participants used a Logitech joystick to perform the tasks during the experiment. The VR setup was implemented using Dassault Systemes 3DVIA Virtools 5.0.

### Visual Stimuli

**Figure 3:**
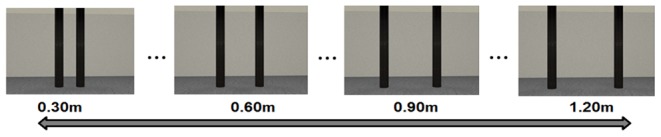
Affordance estimations: participants were able to smoothly adjust the width (from 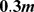 to 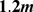) of the gap between the poles. The pictures show the initial size of the gap in each of the four trials.

The virtual scene included a virtual room, a chair, and a curtain behind the chair that was modeled in Autodesk 3ds Max 2010. For the affordance measures we used the two poles (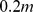 diameter and 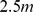 high) from Guess et al. [45] (see Figure 3). The poles cast a bidirectional shadow on the floor of the virtual room. The poles were positioned at a distance of 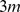 from the participant.

A mesh of a stylized female avatar (from the Rocketbox Studios GmbH: Complete Characters Library HD) was modified in Autodesk 3ds Max 2010 to create the meshes of the underweight and the overweight avatars (see Figure 2). The width of the hips and the shoulders of the underweight avatar were modified to be as thin as possible. Likewise the overweight avatar was modified to be as wide as possible, but still human-like. The width of the hips and the shoulders of the underweight avatar are comparable to those of a female avatar with a body mass index (BMI) of 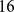. The width of the hips and the shoulders of the overweight avatar are comparable to those of a female avatar with a BMI of 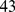.

The 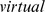 body had the same leg-, arm- and torso-length as the participant. During the entire experiment the 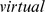 body was seated in the same posture as the participant, with legs together and torso straight, holding a joystick. Thus when looking down at the virtual scene the participants always saw the body of the underweight or the overweight avatar in the same posture as themselves. Note, that in order to track the participant's head motions in our setup the head of the 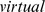 body was not visible. Thus the participants never saw the head of the 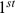 PP 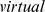 body, nor were they given any additional information about the head of the 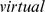 body.

**Figure 4:**
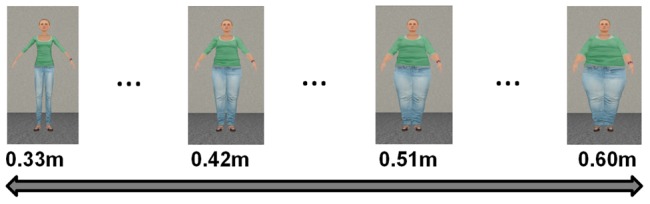
Body size estimations: the participants were able to smoothly adjust the size of the avatar, ranging in width from 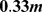 (the underweight avatar) to 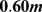 (the overweight avatar). The pictures show the initial size of the 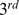 PP avatar in each of the four trials.

For the body size estimation task, the 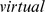 body was shown from 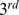 PP (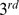 PP avatar) to the participants (see Figure 4). The 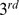 PP avatar also had the same leg-, arm- and torso-length as the participant. Just like the poles in the affordance estimation task, the 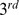 PP avatar was positioned 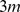 in front of the participants (see Figure 4). To enable smooth adjustment of the body size of the 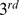 PP avatar we modified the meshes of the underweight and the overweight avatars to create blend shapes (a standard technique in computer animation for changing the shape of objects by interpolating between different meshes) in Autodesk 3ds Max 2010.

To provide the visual stimulation in the virtual scene we used an animated arm of a female character (from the Rocketbox Studios GmbH: Complete Characters Library HD) that was reaching out of a curtain and stroking the legs and arms of the participant (see Figure 2).

### Response measures

For each body type (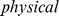, 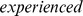 and 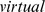) (session I, II and III) we used affordance and body size estimations as response measures (see Figure 3 and Figure 4). Additionally, for the 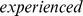 body (session I and II) we used an embodiment questionnaire probing the participants' sense of 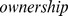 (e.g. "…belongs to me"), 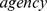 (e.g. "I have control over…") and 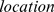 (e.g. "I was sitting in the same location…") (see Table 1). The categories were formed based on the literature and tailored to our specific experiment [7]–[9]. Even though it has been shown [17] that the illusion spreads over the whole body, we were not sure whether this would also be true for a 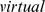 body of a considerably different size than the participant's body. This is why we included ownership and agency questions for each limb separately. The embodiment questionnaire consisted of 34 questions. For each session the questions were listed in randomized order for each participant. The questions used in our experiment are similar to questions used in other body ownership experiments [8], [9], [11], [13], [46], [47]. We used a Likert scale that ranged from 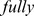 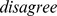 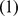 to 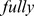 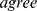 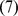. The middle anchor in our questionnaire scale was 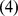 but the participants were not explicitly told that this is the point of uncertainty.

**Table 1:** The list of the items used for the questionnaire in the experiment and its scoring scale.

Sometimes…
…I felt as if the virtual body was my body. (ownership)
…I experienced the virtual body as my body. (ownership)
…I had the feeling that I was looking at myself. (ownership)
…during the experiment I felt heavier than usual. (ownership)
…I experienced the arms of virtual body as parts of myself. (ownership)
…I experienced the legs of virtual body as parts of myself. (ownership)
…I had the feeling that I had a strong connection with the virtual body. (ownership)
…I was not aware that my physical body was different than the virtual body. (ownership)
…it felt as if I had more than one body. (ownership)
…I felt myself somehow connected to the virtual body. (ownership)
…I experienced the virtual body as myself. (ownership)
…it felt like my physical body was changing to take on the shape of the virtual body. (ownership)
…during the experiment I experienced my body bigger than usual. (ownership)
…I had the feeling that the virtual body belonged to me. (ownership)
…during the experiment I felt my physical body had become bigger. (ownership)
…it felt as if the body of the virtual body was my body. (ownership)
…I had the feeling that I and the virtual body were the same. (ownership)
…I had the sensation as if I was feeling the touch at the location at which the left virtual leg was stroked. (location)
…I had the sensation as if I was feeling the touch at the location at which the right virtual leg was stroked. (location)
…it felt like I was feeling touch at the same time as the virtual body was touched. (location)
…it felt as if the touch I was feeling was located somewhere between my physical body and the virtual body. (location)
…I had the feeling that the arm I saw was directly touching me. (location)
…I had the sensation as if I was feeling the touch at the location at which the right virtual arm was stroked. (location)
…I had the sensation as if I was feeling the touch at the location at which the left virtual arm was stroked. (location)
…I had the sensation as though the touch I felt was caused by the arm touching the virtual body. (location)
…I had the feeling that the touch I felt was caused by the arm I saw. (location)
…I had the feeling that I was sitting in the same location as virtual body. (location)
…I felt as if I was inside the virtual body. (location)
…I felt I could move the left arm of the virtual body if I wanted to. (agency)
…I felt I could move the right arm of the virtual body if I wanted to. (agency)
…I felt I could move the right leg of the virtual body if I wanted to. (agency)
…I felt I could move the left leg of the virtual body if I wanted to. (agency)
…I felt I could move the virtual body, if I wanted to. (agency)
…I had the feeling that I had control over the virtual body. (agency)
Requested answer for each item:
Fully disagree O 1 O 2 O 3 O 4 O 5 O 6 O7 fully agree

### Participants

**Figure 5:**
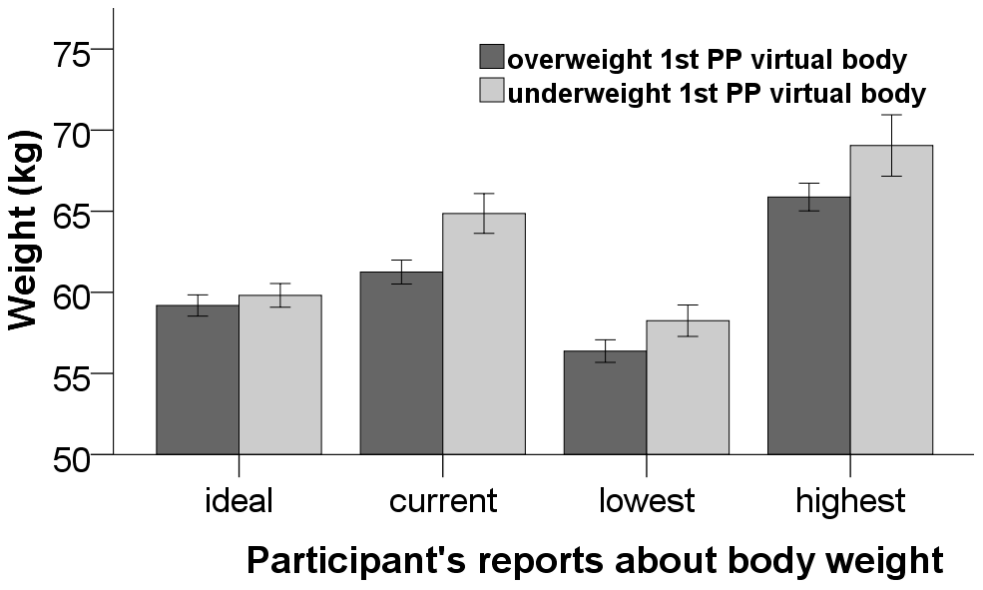
The Graph shows the self-reports of the participants about their ideal, current, lowest (since they were 18 years old) and highest weight (since they were 18 years old). These reports were collected from the EAT-26 and used to screen the participants for eating disorders, in addition to the written consent. Error bars represent one standard error of the mean.

Thirty-two female participants (average age 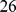 years) with average BMI of 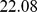, 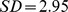, (average weight of 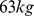, 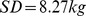, average hip width - 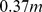, average shoulder width - 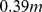) with no history of eating or mental disorders voluntarily participated in our experiment (see Figure 5). The participants were screened for eating disorders in the written consent form of the experiment and with an Eating Attitudes Test^©^  (EAT-26^©^ ). We used only female participants in our experiment mainly because there is a sex difference in the way people perceive the size of their 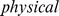 body (see [33] for an overview). Also, since we are manipulating the size of the virtual avatar, the results of our research might be important for research on eating disorders, which shows that young female adults are more likely to engage in disordered eating as compared to young male adults [48], [49]. Each participant was compensated with 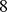 Euros per hour for their participation.

### The Experimental Design

All participants saw a 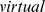 body from 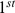 PP, which was visible during the entire experiment. Half of the participants were randomly assigned to the underweight virtual body and the other half of the participants were assigned to the overweight 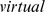 body (see Figure 2). The experiment consisted of three sessions (see Figure 1). The participants had five minute breaks between each session, during which the participant was not wearing the HMD.

Session I differed from session II only in the way the visual-tactile stimulation was provided (synchronous or asynchronous in a counter-balanced order). In both session I and II each participant was instructed to provide estimations for the body she feels she has at the moment (the 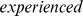 body). In the beginning of session I and II each participant did four trials of affordance estimations followed by four trials of body size estimations. The participant then had one minute (exploration phase - see Table 2) in which she was encouraged to look down and around to get a good image of the body (the 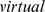 body) and the scene. After that the participant received visual-tactile stimulation. The stimulation was provided for eight minutes in total - stroking both arms and legs for two minutes each, starting with the right upper-arm, then the left upper-arm, followed by the right leg and finally the left leg (see Figure 2 and Table 2). After the visual-tactile stimulation the participant did four trials of affordance estimations followed by four trials of body size estimations. At the end of the session, the participant took off the HMD and using paper and pen answered the embodiment questionnaire (see Table 1). The participants were instructed to give their answers to the questionnaire according to what they felt right after the stimulation.

**Table 2:** The average speed(m/s) with which the participants moved their head during the exploration phase and the visual-tactile stimulation.

visual-tactile stimulation	average head motion speed - overweight virtual body	average head motion speed - underweight virtual body
**no (exploration before synchronous)**	0,010 3 m/s	0,0154 m/s
**no (exploration before asynchronous)**	0,0120 m/s	0,0173 m/s
**right arm synchronous**	0,0034 m/s	0,0035 m/s
**right arm asynchronous**	0,0034 m/s	0,0036 m/s
**left arm synchronous**	0,0037 m/s	0,0048 m/s
**left arm asynchronous**	0,0035 m/s	0,0046 m/s
**right leg synchronous**	0,0030 m/s	0,0036 m/s
**right leg asynchronous**	0,0034 m/s	0,0036 m/s
**left leg synchronous**	0,0032 m/s	0,0036 m/s
**left leg asynchronous**	0,0034 m/s	0,0036 m/s

Session III consisted of two parts. During the first part of session III each participant was instructed to ignore the visual information from the 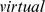 body and to do four trials of affordance followed by four trials of body size estimations of her 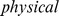 body. In the second part of session III each participant was asked to ignore the information (e.g. sensory, memory) that she perceives from her 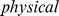 body and to perform four trials of affordance followed by four trials of body size estimations of the 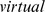 body.

At the end of the experiment each participant was debriefed, filled in an EAT-26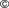 in which they reported their current, their lowest and their highest weights and the experimenter measured the width of their hips and shoulders. In total, the experiment, including breaks between sessions, took about two hours.

### Preparation for the experiment

Before the beginning of the experiment each participant was given written and oral instructions by the experimenter. Then the participant's height, arm- and leg-lengths were measured for scaling both the 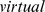 body and the 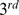 PP avatar used for the body size estimation task. The participant then sat on a chair with her legs together and torso straight, holding a joystick in her hands. The participant put on the HMD and was asked to look down to report whether she sees a virtual joystick (the virtual joystick was positioned in the hands of the 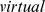 body). Thus the participant's attention was indirectly pointed to the 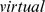 body. This was to make sure that each participant saw the 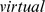 body before performing the tasks.

### Affordance estimation procedure

Our affordance estimation task consisted of four trials, in which the participant adjusted the distance between two poles to an aperture size that would allow the target body (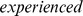, 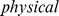 or 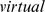) to pass through without twisting the shoulders or hips. The participants used a joystick to smoothly move the poles in either direction (step-size - 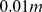) (see Figure 3). The initial widths of the gap were 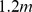, 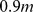, 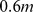 or 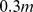 (see Figure 3). Each width was presented in one trial in randomized order.

### Body size estimation procedure

The body size estimation task consisted of four trials, in which the participant used the joystick to adjust the body size of the 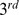 PP avatar to match the target body (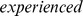, 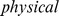 or 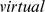) (see Figure 4). Participants could smoothly adjust the body size of the 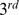 PP avatar to the desired size/shape (step-size - 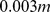). For the body size estimation task we used as starting points variations of the 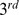 PP avatar, in which the widest part was 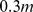, 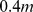, 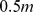 or 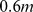 (see Figure 4). Each was presented in one trial in randomized order.

### Visual-tactile stimulation procedure

The experimenter provided tactile stimulation by stroking (with her hand) the participant, either synchronously or asynchronously with the visual stimulation. During visual-tactile stimulation, the participants were asked to look at the direction of the limb that was being stimulated, and not to move their head (see Table 2). The experimenter made sure that the participant always had the limb in view by not starting the session until the participant was looking at the limb and by encouraging the participant to look at the limb being stroked at all times (see Table 2). Visual-tactile stimulation was provided through stroking for eight minutes in total - starting from the right upper-arm, then the left upper-arm, followed by the right leg and finally the left leg (see Figure 2). Visual stimulation was provided by a virtual arm coming out of the curtain. The experimenter provided tactile stimulation to the corresponding limb of the participant.

## Results

### Analysis of the questionnaires

**Figure 6:**
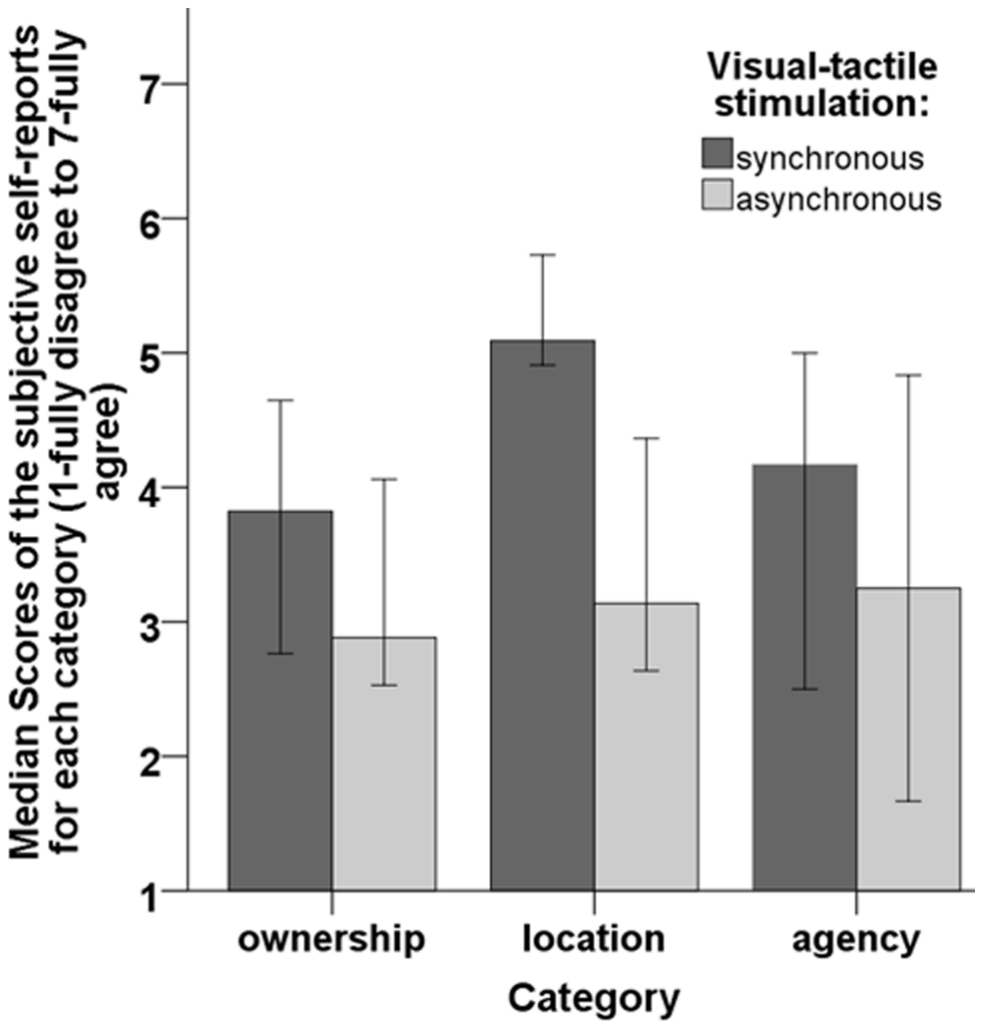
Graph showing the median score of the subjective self-reports organized into categories. Error bars represent 95% confidence intervals of the median.

We analyzed the answers from the categories (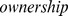, 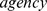 and 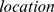) of the embodiment questionnaire using Wilcoxon signed-rank tests with planned comparisons, because the responses from the ownership category in the synchronous session and the responses from the agency category in the asynchronous session from the overweight condition were not normally distributed, (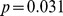 and 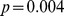 respectively). The homogeneity of variances was only violated for 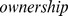 for both the synchronous and the asynchronous stimulation, (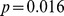 and 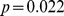 respectively), for the rest 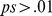. The analysis showed that significantly greater levels of embodiment were observed after the session with the synchronous compared to the asynchronous visual-tactile stimulation: ownership, (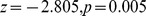), location, (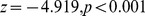) and agency, (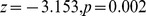) (see Figure 6). There was no significant difference between the levels of subjective ownership between the group that saw the underweight body and the group that saw the overweight body (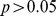).

### Analysis of the affordance and the body size estimations

We analyzed the affordance and body size estimations to investigate whether the sense of body ownership over the 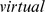 body (for both the underweight and the overweight) also had an influence on the participants' perceived body dimensions. Since we used a stylized avatar we could not precisely measure the BMI of each mesh. Therefore, we based our estimations on the widest body part of the participants and the 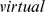 body. In our case this was either the width of the hips or the width of the shoulders. These are also the measurements that are most relevant for the affordance estimations. For analyzing the effect of the 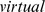 body on the participant's perception, we calculated the ratio of the affordance estimations and the body size estimations based on the actual width of the participants. For calculating the width (both actual and estimated) we always considered the width of the widest body part (the hips or the shoulders):
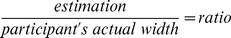
(1)


#### Affordance estimations

**Figure 7:**
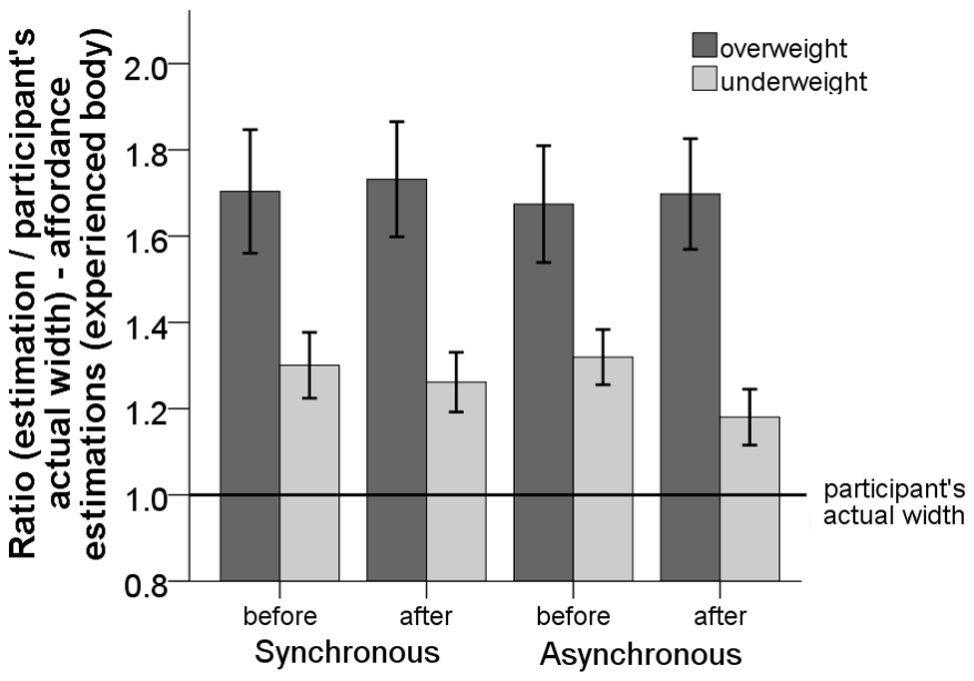
Plot of the ratio of the affordance estimations for the 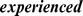 body before and after synchronous and asynchronous visual-tactile stimulation. Error bars represent one standard error of the mean.

We performed a three-way mixed repeated measures ANOVA with the visual-tactile stimulation (synchronous vs. asynchronous) and the estimation (before vs. after) as within subject factors, the size of the 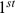 PP 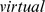 body (underweight vs. overweight) as a between subject factor, and the ratio of the affordance estimations (normally distributed - Shapiro-Wilk test 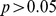, the homogeneity of variances was not violated after transformation 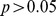) as a dependent variable (see Figure 7). The ANOVA showed a significant main effect of the size of the 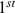 PP 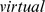 body (overweight - 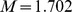, 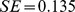; underweight - 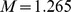, 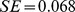) on the ratio of the affordance estimations, (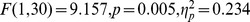). The stimulation did not have an effect on the ratio of the affordance estimations, (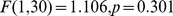). The estimation (before vs. after) did not have an effect on the ratio of the affordance estimation, (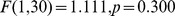). There was no significant interaction between the visual-tactile stimulation and the size of the 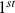 PP virtual body (underweight vs. overweight), (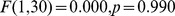), nor an interaction between the size of the 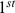 PP virtual body and the estimation (before vs. after), (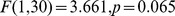).

#### Body size estimations

**Figure 8:**
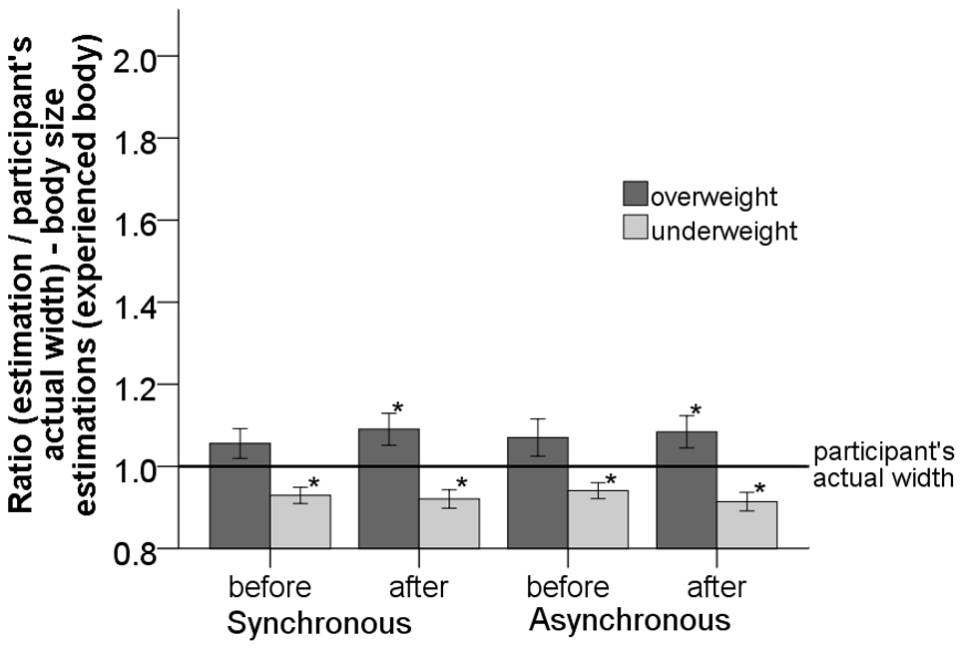
Plot of the ratio of body size estimations for the 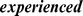 body before and after synchronous and asynchronous visual-tactile stimulation. The asterisk (*) shows the estimations that are significantly different from the participant's actual width. Error bars represent one standard error of the mean.

The body size estimations for the 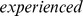 body were analyzed using a three-way mixed repeated measures ANOVA with visual-tactile stimulation (synchronous vs. asynchronous) and the order of estimation (before vs. after) as within subject factors, the size of the 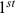 PP virtual body (underweight vs. overweight) as a between subject factor and the ratio of body size estimations (normally distributed - Shapiro-Wilk test 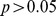, the homogeneity of variances was not violated after transformation, 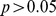) as a dependent variable (see Figure 8). The analysis showed a significant main effect of the size of the 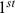 PP virtual body (overweight - 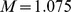, 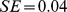; underweight - 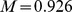, 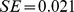) on the ratio of body size estimations, (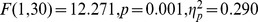). The stimulation (synchronous vs. asynchronous) did not have a significant main effect on the ratio of body size estimations, (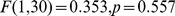). The order of estimation (before vs. after) did not have an effect on the ratio of the body size estimation, (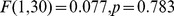). There was no significant interaction between the visual-tactile stimulation and the size of the 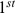 PP virtual body (underweight vs. overweight), (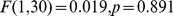), nor an interaction between the size of the 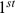 PP virtual body and the estimation (before vs. after), (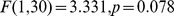).

#### The effect of visual stimuli

We further investigated whether the visual perception of the size of the 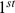 PP virtual body (underweight vs. overweight) had an effect on the estimations even before the visual-tactile stimulation. Using a paired-samples t-test we compared the ratio of the actual width (actual participant's width / actual participant's width  =  ratio of the actual width) to the ratio of the affordance or the body size estimation before the stimulation. The t-test showed that the affordance estimations for the 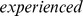 body were significantly underestimated (for the underweight 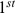 PP virtual body) and overestimated (for the overweight 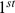 PP virtual body) compared to the participant's actual width (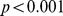) even before stimulation (see Figure 7). Interestingly, before the visual-tactile stimulation the body size estimations for the 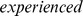 body were significantly underestimated (for the underweight 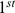 PP virtual body: 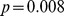 - asynchronous; 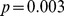 - synchronous), but not significantly overestimated (for the overweight 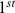 PP virtual body: 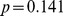 - asynchronous; 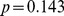 - synchronous) compared to the participant's actual width (see Figure 8). Additionally, the head-tracking data indicated that participants who saw the overweight 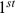 PP virtual body moved their heads less during the exploration phase and the visual-tactile stimulation as compared to participants who saw the underweight virtual body (see Table 2).

#### Correlations

We investigated the relationship of the mean values between the ratios of the subjective self-reports (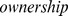, 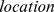 and 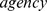) and the behavior measures (body size and affordance estimations) provided for each of the target bodies (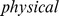, 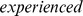 and 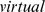), as well as the relationship between ratios of the participants' actual body size and the response measures (subjective self-reports, body size and affordance estimations). The analysis revealed several significant relationships between ratios of the subjective self-reports and the behavior measures in the overweight condition and in the underweight condition (see Table 3). Additionally, we found significant correlations between ratios of the actual size of the participants and the response measures in the overweight condition and a significant correlation between the actual size of the participants and the body size estimations in the underweight condition (see Table 3).

**Table 3:** The correlations between the ratios of the subjective self-reports, the behavioral response measures (body size and affordance estimations) and participants' measurements for both the underweight (u) and the overweight (o) conditions.

		Subjective self-reports (combined)			Affordance estimations			Body size estimations			Participants' measurement (width)		
		Ownership	Location	Agency	Physical body	Exper. body	Virtual body	Physical Body	Exper. body	Virtual body	Hips	Shoulders	Widest body part
**Subjective self-reports (combined)**	**Ownership**		u = .747** o = .705**	u = .773** o = .603**									
	**Location**	u = .747** o = .705**		o = .856**				o = −.395*					
	**Agency**	u = .773** o = .603**	o = .856**				o = −.443*	o = −.445*		o = −.588**		o = .565**	o = .565**
**Affordance estimations**	**Physical body**					u = .888** o = .826**	o = .886**	o = .440*	u = .488* o = .461*	o = .427*			
	**Exper. body**				u = .888** o = .826**		u = .529* o = .835**		u = .529* o = .778**	o = .404*	o = .465*		
	**Virtual body**			o = −.443*	o = .886**	u = .529* o = .835**			o = .458*	u = .521* o = .612**		u = −.624**	
**Body size estimations**	**Physical body**		o = −.395*	o = −.445*	o = .440*				u = .771** o = .494**	u = .555* o = .456*		o = −.604**	o = −.604**
	**Exper. body**				u = .488* o = .461*	u = .529* o = .778**	o = .458*	u = .771** o = .494**		u = .675** o = .394*	o = .370*		
	**Virtual body**			o = −.588**	o = .427*	o = .404*	u = .521* o = .612**	u = .555* o = .456*	u = .675** o = .394*			u = −.693** o = −.829**	u = −.760** o = −.829**
**Participants**' **measurement (width)**	**Hips**					o = .465*			o = .370*				u = .712**
	**Shoulders**			o = .565**			u = −.624**	o = −.604**		u = −.693**, o = −.829**			u = .880** o = 1.000**
	**Widest body part**			o = .565**				o = −.604**		u = −.760** o = −.829**	u = .712**	u = .880** o = 1.000**	

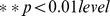
 **,** 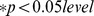 **, overweight (o) (** 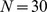 **), underweight (u) (** 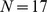 **)**

### Results - experienced body, physical body and virtual body

In contrast to sessions I and II, in session III participants did not receive visual-tactile stimulation (see Figure 1). In session III participants provided affordance and body size estimates about their 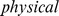 body and their 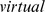 body. The dependent measures for the 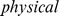 body were obtained first, then the participants provided the measures for the 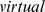 body.

Considering that in session I and II the visual-tactile stimulation did not have an effect on the affordance and body size estimations, we combined the estimations from session I and II. To gain more insight about the influence of the virtual body on the participants' perception of affordances and body size, we compared the estimations provided for the 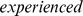 body to the estimations provided for the 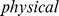 body and the 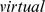 body.

#### Affordance estimations

**Figure 9:**
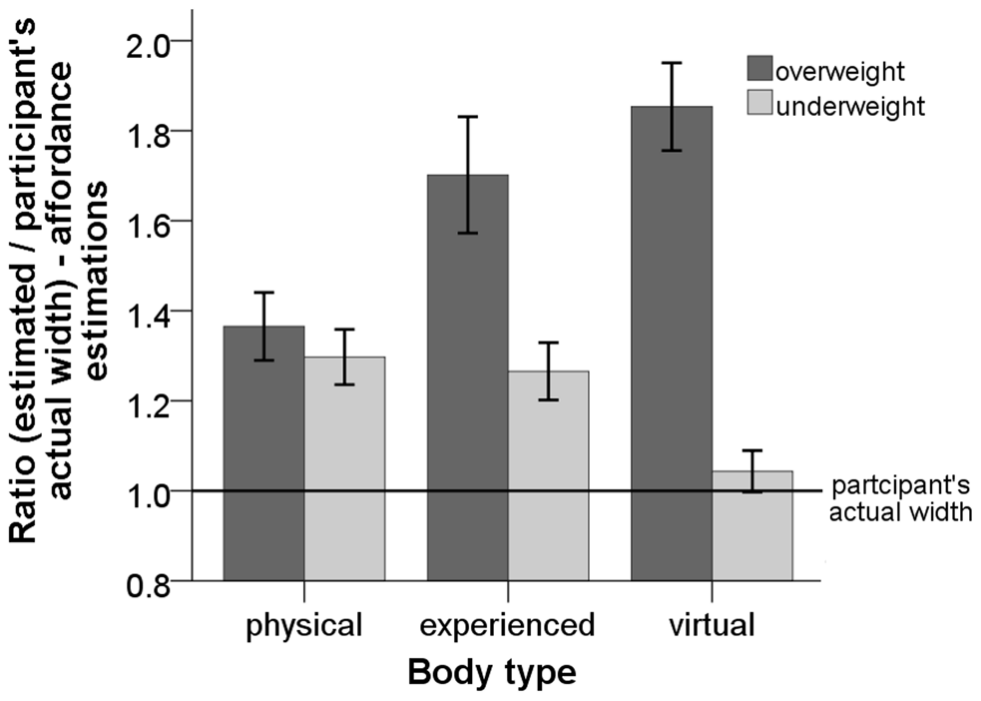
Plot of the ratio of the affordance estimates for the 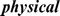, 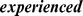 and 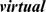 bodies. Error bars represent one standard error of the mean.

We performed a two-way mixed repeated measures ANOVA (and post hoc tests) with body type (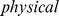 vs. 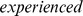 vs. 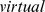) as a within subject factor, the size of the 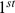 PP virtual body (underweight vs. overweight) as a between subject factor, and the ratio of the affordance estimations as a dependent variable (the homogeneity of variances was not violated after transformation, 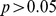) (see Equation 1 and Figure 9). The analysis revealed a significant main effect of the size of the 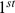 PP virtual body (underweight vs. overweight) on the ratio of the affordance estimations, (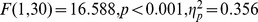). The body type (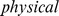 vs. 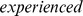 vs. 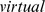) had a significant main effect on the ratio of the affordance estimations (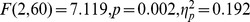). The analysis also showed a significant interaction between body type and the size of the 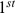 PP virtual body, (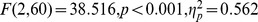).

For the underweight 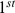 PP virtual body the pairwise comparison using LSD adjustment for multiple comparison showed that at the 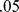 level of significance the ratio of the affordance estimates provided for the 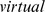 body (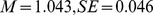) was significantly underestimated compared to the ratio of the affordance estimates for the 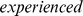 body (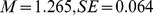) (p = 0.001) and the 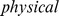 body (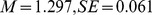) (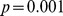). All the other comparisons were not significant.

For the overweight 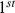 PP virtual body the pairwise comparison using LSD adjustment for multiple comparison showed that at the 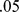 level of significance the ratio of the affordance estimates provided for the 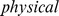 body (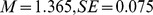) were significantly underestimated compared to the ratio of the affordance estimates for the 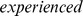 body (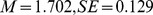) (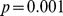) and the 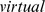 body (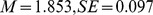) (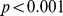).

Additionally, we investigated the effect of the size of the 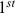 PP 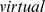 body on the estimates for the 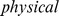 body. Therefore, we performed a paired-samples t-test to compare the ratios of the affordance estimates for the 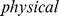 body when visual sensory input of an underweight 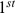 PP 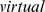 body was provided to when visual sensory input of an overweight 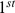 PP 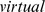 body was provided. The t-test reveals that the ratio of the affordance estimates for the 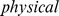 body are not statistically significant (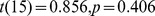).

#### Body size estimations

**Figure 10:**
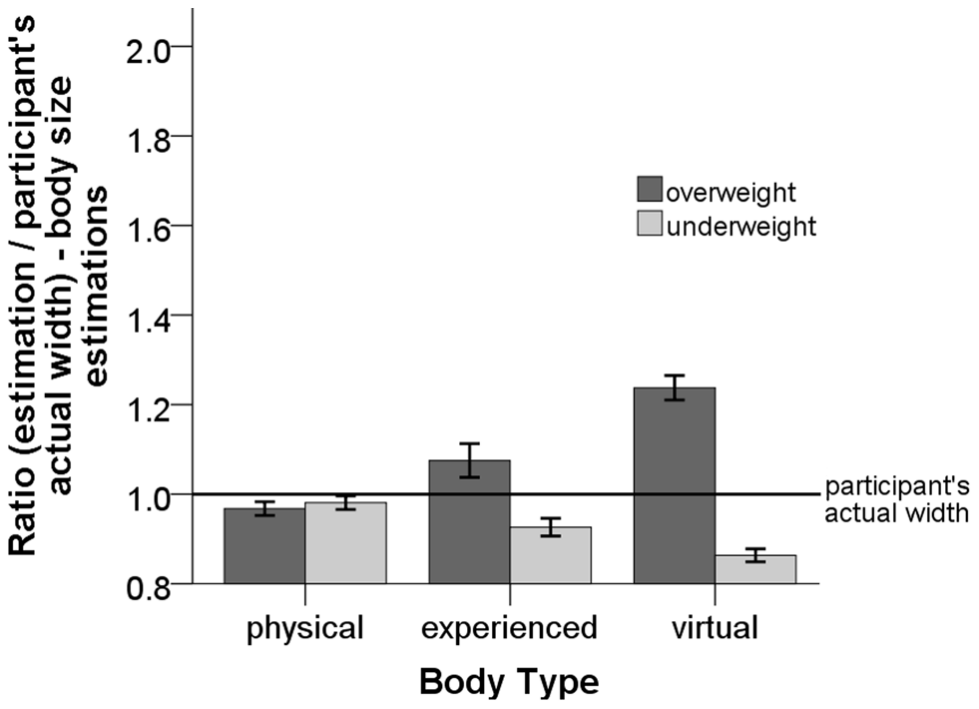
Plot of the ratio of the body size estimates for the 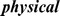, 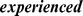 and 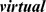 bodies. Error bars represent one standard error of the mean.

We performed a two-way mixed repeated measures ANOVA with the body (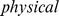 vs. 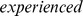 vs. 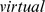) as a within-subject factor, the size of the 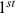 PP 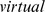 body (underweight vs. overweight) as a between-subject factor, and the ratio of the body size estimates as a dependent variable (the homogeneity of variances was not violated after transformation, 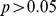) (see Equation 1 and Figure 10). The size of the 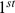 PP 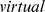 body (underweight vs. overweight) had a significant main effect on the ratio of body size estimates, (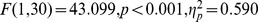). The analysis also revealed that the body type (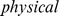 vs. 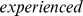 vs. 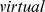) had a significant main effect on the ratio of the body size estimates, (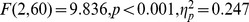). The interaction between the body type and the size of the 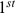 PP 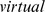 body (underweight vs. overweight) was also significant, (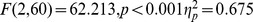).

For the underweight 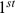 PP 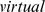 body the pairwise comparison using LSD adjustment for multiple comparison showed that at the 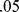 level of significance the ratio of the body size for the 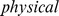 body (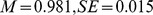) was significantly overestimated compared to the 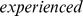 body (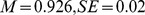) (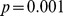). The experienced body was significantly overestimated compared to the 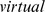 body (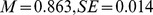) (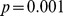). The 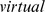 body was significantly underestimated compared to the 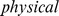 body (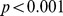).

For the overweight 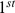 PP 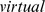 body the pairwise comparison using LSD adjustment for multiple comparison showed that at the 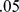 level of significance the ratio of the body size estimations for the 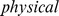 body (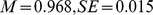) was significantly underestimated compared to the experienced body (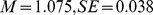) (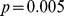). The experienced body was significantly underestimated compared to the 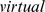 body (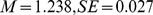) (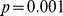). The 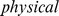 body was also significantly underestimated compared to the 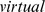 body (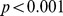).

Further, we investigated the effect of the size of the 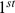 PP 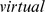 body on estimations of the 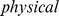 body. We used a paired-samples t-test to compare the ratios of the body size estimates for the 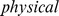 body from the underweight 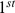 PP 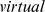 body to the ratio of the body size estimates for the 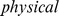 body from the overweight 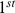 PP virtual body. The t-test showed that the body size estimates for the 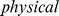 body are not statistically different (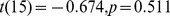).

After session III the participants were asked whether and when they felt that the 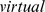 body was representing them in the VE. Eight participants reported that they felt represented by the 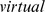 body during both the sessions with synchronous and asynchronous stimulation. Seventeen participants felt represented by the 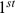 PP 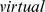 body only during the session with synchronous stimulation, while the remaining seven participants answered that they did not feel represented by the 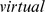 body (five - overweight 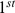 PP 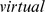 body, two - underweight 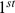 PP 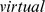 body).

## Discussion

### Discussion - questionnaires

The subjective self-reports provided by our participants indicated that after synchronous visual-tactile stimulation women experienced a significantly stronger sense of body ownership compared to after the asynchronous stimulation. Our results are consistent with the findings from RHI paradigms employed in both the VR [10]–[12], [14]–[16] and the real world [4]–[7], [13], [50]. It is interesting that the participants are not completely rejecting the virtual body after the asynchronous stimulation by providing responses around 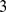 for each of the three categories (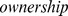, 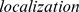 and 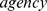) and thus, suggesting that the synchronous head movements, the congruent visual perspective and the posture may have played a role in the illusion and were not negatively affected by the asynchronous visual-tactile feedback. Therefore, head motion and 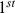 PP may have partly overridden asynchronous touch. In our setup participants first estimated their affordances and body size, then answered the embodiment questionnaire, while in the most experiments using a RHI paradigm participants answer a questionnaire right after the stimulation. It is possible that our participants gave lower scores for the questions than what they would report if the questionnaire were answered right after the stimulation.

Interestingly, our results also showed that participants experienced not only a sense of ownership over the limbs of the virtual body (underweight or overweight) which spread to the entire body, but also a sense of self-localization and a sense of agency. Probably, the sense of self-localization was induced due to the visual stimulus of the 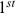 PP virtual body. Note that in RHI paradigms employed in the real world the sense of agency can only be induced when in addition to the visual and the proprioceptive feedback, active synchronous sensory-motor feedback is provided [7], [9], [50]. So far, researchers who have used 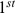 PP virtual avatars and employed the RHI paradigm in VR did not use questions related to agency [10], [11], [16]. However, our findings hint that in VR setups similar to ours, the sense of agency is induced without providing active synchronous sensory-motor feedback from the virtual body or its limbs. Furthermore, it seems that even though the head tracking was from the participants own head motions, participants interpreted cues from head tracking as if they were provoked by the head motions of the virtual body, therefore the participants felt a sense of agency over the body. Thus, the cues provided by head tracking were probably perceived as active synchronous sensory-motor feedback, although it was not the head motion of the virtual body. This suggests that the illusion can be spread from a body part to the whole body, as found in Petkova et al. [17], but our results indicate that this can even include the out of view head of the avatar. It is possible that the proprioceptive feedback from the participant's 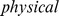 body and the cues provided by head tracking combined with the visual stimuli (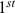 PP virtual body in a similar posture) were enough to influence the participant's sense of agency over the virtual body. However, further research on this topic is necessary.

### Discussion - affordance and body size estimations

We found that the size of the 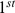 PP virtual body (underweight vs. overweight) significantly biased the participants' affordance and body size estimations of the 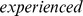 body. Interestingly, even though the synchronous visual-tactile stimulation had a significant influence on the participants' self-reports, it did not significantly impact the ratio of the participants' affordance and body size estimations. It is possible that the subjective self-reports and the behavioral measures (such as body size and affordance estimations) are influenced by different stimulation strategies. Therefore, in order to influence the affordance and body size estimations it might be preferable to use another type of stimulation or employ different methods for performing the behavioral measures. Another potential explanation for why we found no significant effect of the stimulation on the behavioral measures is provided by the participants' reports collected before visual-tactile stimulation. Contrary to our expectations, our results showed that participants experienced a significant change in their 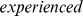 body size (only for the underweight) and affordances (for both) even before any type of visual-tactile stimulation. Considering the research of Normand et al. [11] we think that the lack of effect of the stimulation on the body size estimations after the synchronous stimulation suggests that women might be more susceptible to visual stimulation and felt larger/smaller even before the tactile stimulation. However, further investigation is necessary in order to have conclusive results as to whether the difference between our results and the findings of Normand et al. [11] could be explained by sex differences or other differences in the experimental setup, especially since a recent study by Preston et al. [47] reported no sex difference in a body size manipulation task involving a similar RHI paradigm. Preston et al. [47] found that illusory ownership over a large body does not have an effect on the perceived body width, while ownership over a slimmer body caused a significant decrease in the participant's perceived body width. Our results from the body size and affordance estimations tasks confirm the findings of Preston et al. [47] about perceived body size when experiencing illusory ownership over a large body. However, we found no effect of stimulation for body size and affordance estimations tasks also for the thinner body. It is possible that the differences in the setup (e.g. VE, body size estimation procedure) caused the difference between our results and the findings reported in Preston et al. [47] about the thin body.

Interestingly, our results from body size estimations relate to recent research in which the participants estimated that distorted photographs (reduced by 10% in width) of their own bodies were their current body size, while photographs that were increased by 30% in width were often judged as belonging to others [51]. Note, that in their experiment, Hashimoto and Iriki, 2013 [51], do not apply a RHI paradigm. Moreover, considering the related literature which employs the RHI paradigm, it is unlikely that our participants experienced full body ownership over the 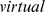 body only after looking at it for 0.3 min without experiencing any type of synchronous visual-tactile or sensory-motor stimulation. However, it is possible that in order to influence the participant's body size and affordance estimations it is not necessary to use all the cues (e.g. a congruent position of the rubber hand with the participant's real hand [6], [20], visual-tactile stimulation [6]–[16], [20], [50]) necessary for inducing body ownership.

Our findings suggest that a combination of several congruent cues was enough to influence participants' perception of affordances and body size even before any visual-tactile stimulation. These cues, namely cues perceived from head tracking, visual cues (the underweight/overweight virtual body, which was viewed from 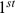 PP sitting in the same posture as the participant) and somatosensory stimulation (provided from the participant's 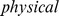 body, which they were instructed not to move) were enough to influence participants' perception of body size (only for the underweight condition) and affordances. Perhaps another crucial factor for the effect of the 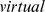 body on the 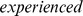 body size and affordance estimations was the method we used for conducting the affordances and the body size estimations. However, our participants provided self-reports only after the visual-tactile stimulation, therefore we have no evidence for whether (in addition to the experienced change in body size and affordances) they experienced a sense of ownership over the 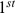 PP virtual body even before the visual-tactile stimulation.

### Discussion - distinction between the physical, virtual and experienced body

We made a distinction between the 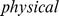, 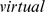 and 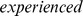 body to gain a better understanding of which cues (e.g. visual, proprioceptive, memory or a combination of these) had greater impact on body perception. This methodological point is important for VR experiments (that employ the RHI paradigm) which aim to investigate the specific cues that influence body perception. The affordance and body size estimations indicated that participants perceived the three body types (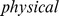, 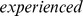 and 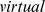) differently. Even though the 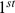 PP virtual body was visible during the entire experiment, the estimates for the 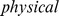 body were veridical. Participants probably based their estimates for the 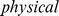 body on their memory and the proprioceptive information they perceived from their 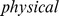 body, and ignored the visual stimuli (as instructed).

The affordance and body size estimates for the overweight 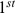 PP 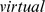 body were significantly overestimated compared to the estimates for the underweight 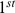 PP virtual body. Interestingly, our findings suggest that in estimating the 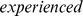 body, our participants integrated the information perceived from visual stimuli in the VE (e.g. the 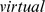 body shown from 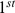 PP and head tracking) with the proprioceptive and somatosensory information from their 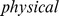 body. Another potential reason might be that the participants were confused by the mismatch between the visual cues that they perceived from the VE and other cues, such as memory and proprioception.

## Conclusion

Using subjective self-reports, we showed that after synchronous, visual-tactile stimulation, women experience a significantly stronger agreement (as compared to after asynchronous stimulation) with the statements of an embodiment questionnaire probing the participant's sense of 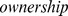, 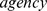 and 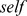-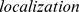 with respect to a virtual body of a considerably different size than the participant's 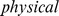 body. In contrast to other researchers who have used 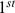 PP virtual avatars and employed the RHI paradigm in VR, in our study we used questions related to agency. Interestingly, the participants experienced a sense of agency over the virtual body and its limbs, even though no active synchronous sensory-motor feedback from the virtual body or its limbs was provided. Our findings suggest that the participant's sense of agency is influenced by the combination of proprioceptive and somatosensory feedback from the 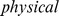 body and the cues provided from the head tracking and the visual stimuli (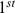 PP 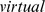 body in similar posture). It is possible that participants are trying to integrate the contrasting stimuli that they perceive from visual stimuli in the VR and proprioceptive information from their body into one coherent percept.

In addition to traditional methods using embodiment questionnaires, we quantified body ownership by introducing new behavioral response measures, namely, affordance (indirect measure of body size) and body size (direct measure of body size) estimations. Though we did not find an effect of visual-tactile stimulation on the affordance and body size estimations, our analysis showed that even before visual-tactile stimulation our participants perceived a change in their 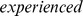 body dimensions (consistent with the size of the virtual body). Another important point introduced in our study was the distinction between the 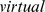, 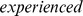 and 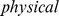 body. Participants' affordance and body size estimates show that the three body types were perceived differently. The affordance and body size estimates for the 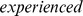 body were influenced by visual stimuli (the size of the 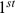 PP 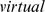 body), whereas the affordance and body size estimates for the 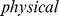 body show that, if instructed, participants can dissociate visual feedback from perceived proprioceptive feedback and memory of their 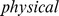 body.

Our results have several important implications for setups which aim to influence cues related to participants' perception of body size and ownership. Since we are using a measure which enables the participants to precisely adjust the size of the virtual body, our findings could be of interest to researchers who are developing new strategies for using VE applications for therapies to help patients with body image disorders. It is not necessary for an avatar representing the participant in the HMD VE to have the same size (in terms of weight) as the participant, it is sufficient to provide sensory feedback, such as a 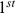 PP virtual body (in a similar posture to the participant) and head tracking. Providing sensory (e.g. visual stimuli, head tracking, somatosensory information) feedback similar to that provided in our experimental setup is enough to induce a sense of agency over the virtual body. The distinction between the three body types (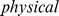, 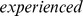 and 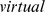) is an important methodological point for VR experiments (that employ the RHI paradigm). It can be used to collect estimations based on specific cues that influence body perception. Thus one can have a better understanding of which cues (e.g. visual, proprioceptive, somatosensory, memory or a combination thereof) have a greater impact on body perception and whether it varies between different setups.
